# Lung cancer in systemic lupus erythematosus

**DOI:** 10.1186/ar3949

**Published:** 2012-09-27

**Authors:** M Kale, R Ramsey-Goldman, S Bernatsky, MB Urowitz, D Gladman, PR Fortin, M Petri, E Yelin, S Manzi, S Edworthy, O Nived, S-C Bae, D Isenberg, A Rahman, JG Hanly, C Gordon, S Jacobsen, E Ginzler, DJ Wallace, GS Alarcón, MA Dooley, L Gottesman, K Steinsson, A Zoma, J-L Senécal, S Barr, G Sturfelt, L Dreyer, L Criswell, J Sibley, JL Lee, AE Clarke

**Affiliations:** 1 McGill University Health Centre, Montreal, QC, Canada; 2 Northwestern University Feinberg School of Medicine, Chicago, IL, USA; 3 Toronto Western Hospital, Toronto, ON, Canada; 4 Université de Laval, QC, Canada; 5 Johns Hopkins University School of Medicine, Baltimore, MD, USA; 6 University of California, San Francisco, CA, USA; 7 West Penn Allegheny Health System, Pittsburgh, PA, USA; 8 University of Calgary, AB, Canada; 9 Lund University Hospital, Lund, Sweden; 10 The Hospital for Rheumatic Diseases, Hanyang University, Seoul, Korea; 11 University College, London, UK; 12 Dalhousie University and Capital Health, Halifax, NS, Canada; 13 College of Medical and Dental Sciences, University of Birmingham, UK; 14 Rigshospitalet, Copenhagen University Hospital, Copenhagen, Denmark; 15 State University of New York - Downstate Medical Center, Brooklyn, NY, USA; 16 Cedars-Sinai Medical Center/David Geffen School of Medicine, University of California Los Angeles, CA, USA; 17 The University of Alabama, Birmingham, AL, USA; 18 University of North Carolina at Chapel Hill, NC, USA; 19 Landspitali University Hospital, Reykjavik, Iceland; 20 Lanarkshire Centre for Rheumatology, Hairmyres Hospital, East Kilbride, UK; 21 Université de Montréal, QC, Canada; 22 Copenhagen University Hospital, Copenhagen, Denmark; 23 Royal University Hospital, Saskatoon, Saskatchewan, Canada

## Background

An increased lung cancer risk in SLE has been suggested in the literature [1]. Our objective was to provide an updated analyses of the lung cancer cases from a multi-site international cohort study, including descriptive statistics of the demographics (age, sex, and race/ethnicity) of cases, as well the of histology.

## Methods

Data from an SLE sample of 15,980 SLE patients from 28 centers were analyzed (in Canada, the United States, the United Kingdom, Denmark, Sweden, Scotland, Korea and Iceland). Information on date of birth, sex and race was available, along with the date of SLE diagnosis. Cancer occurrence was ascertained through linkages with regional tumor registries. We assessed the demographic characteristics for all lung cancer cases in the SLE patients, and information on histology types was analyzed from the centers where this information was available.

## Results

In the current analyses, 101 lung cancer cases that had occurred after SLE diagnosis were studied. The lung cancer cases were distributed across 21 centers. The average age of the SLE patients at lung cancer diagnosis was 60 years (median 40, standard deviation (SD) 10.9). The average SLE duration at the time of lung cancer diagnosis was 13 years (median 12, SD 10.6). Race/ethnicity was not provided by six centers (35 cases). Of the remaining 66 cases, the majority were Caucasian (*n *= 54, 53.5%) followed by nine African-American, one Asian, one Pacific-Islander and one of unknown racial/ethnic origin. Histological lung cancer type was only provided by 12 centers (59 cases). The most common histological type reported within these 59 cases was squamous cell carcinoma (*n *= 15, 25.4%; 95% CI = 16.1 to 37.8) followed by adenocarcinoma (*n *= 13, 22%; 95% CI = 13.4 to 34.1) and nonsmall-cell carcinoma (*n *= 5, 8.5%; 95% CI = 3.7 to 18.4). The remaining 44% were composed of carcinomas not otherwise specified and a variety of uncommon tumors: large cell, clear cell, solid, bronchoalveolar, adenosquamous, epithelial hemangioendothelioma, oat cell, carcinoid, small cell and mucinous histological types.

## Conclusion

In the general population, about 30 to 40% of lung cancer cases are adenocarcinoma, with 20 to 30% squamous cell carcinoma, and 10% large cell carcinoma [2]. Our results suggest a similar distribution, but with a possibly lower proportion of adenocarcinomas, and a higher number of uncommon lung cancer types. Further work is planned to assess other features of these cancers.
